# Revealing the Virulence Potential of Clinical and Environmental *Aspergillus fumigatus* Isolates Using Whole-Genome Sequencing

**DOI:** 10.3389/fmicb.2019.01970

**Published:** 2019-09-04

**Authors:** Fabiola Puértolas-Balint, John W. A. Rossen, Claudy Oliveira dos Santos, Monika M. A. Chlebowicz, Erwin C. Raangs, Maarten L. van Putten, Pedro J. Sola-Campoy, Li Han, Martina Schmidt, Silvia García-Cobos

**Affiliations:** ^1^University of Groningen, University Medical Center Groningen, Department of Medical Microbiology and Infection Prevention, Groningen, Netherlands; ^2^University of Groningen, Department of Molecular Pharmacology, Groningen, Netherlands; ^3^Reference and Research Laboratory on Antimicrobial Resistance and Healthcare Infections, National Microbiology Centre, Institute of Health Carlos III, Madrid, Spain; ^4^Institute of Disease Control and Prevention, Academy of Military Medical Sciences, Beijing, China; ^5^University Medical Center Groningen, Groningen Research Institute for Asthma and COPD, Groningen, Netherlands

**Keywords:** *Aspergillus fumigatus*, virulence, whole-genome sequencing, clinical and environmental isolates, gene database

## Abstract

*Aspergillus fumigatus* is considered a common causative agent of human fungal infections. A restricted number of virulence factors have been described, and none of them lead to a differentiation in the virulence level among different strains. Variations in the virulence phenotype depending on the isolate origin, measured as survival percentage in animal infection models, have been previously reported. In this study, we analyzed the whole-genome sequence of *A. fumigatus* isolates from clinical and environmental origins to determine their virulence genetic content. The sample included four isolates sequenced at the University Medical Center Groningen (UMCG), three clinical (two of them isolated from the same patient) and the experimental strain B5233, and the draft genomes of one reference strain, two environmental and two clinical isolates obtained from a public database. The fungal genomes were screened for the presence of virulence-related genes (VRGs) using an in-house database of 244 genes related to thermotolerance, resistance to immune responses, cell wall formation, nutrient uptake, signaling and regulation, and production of toxins and secondary metabolites and allergens. In addition, we performed a variant calling analysis to compare the isolates sequenced at the UMCG and investigated their genetic relatedness using the TRESP (Tandem Repeats located within Exons of Surface Protein coding genes) genotyping method. We neither observed a difference in the virulence genetic content between the clinical isolates causing an invasive infection and a colonizing clinical isolate nor between isolates from the clinical and environmental origin. The four novel *A. fumigatus* sequences had a different TRESP genotype and a total number of genetic variants ranging from 48,590 to 68,352. In addition, a comparative genomics analysis showed the presence of single nucleotide polymorphisms in VRGs and repetitive genetic elements located next to VRG groups, which could influence the regulation of these genes. In conclusion, our genomic analysis revealed a high genetic diversity between environmental and clinical *A. fumigatus* isolates, as well as between clinical isolates from the same patient, indicating an infection with a mixed-population in the latter case. However, all isolates had a similar virulence genetic content, demonstrating their pathogenic potential at least at the genomic level.

## Introduction

*Aspergillus fumigatus* is an opportunistic fungal pathogen that poses major threats to immunocompromised individuals in clinical settings. High-risk patients include neutropenic patients, hematopoietic stem cell transplant recipients, patients receiving prolonged steroid treatment, and critically-ill patients in the intensive care unit (ICU) with chronic obstructive pulmonary disease (COPD), liver cirrhosis, viral infections, or microbial sepsis (Hohl and Feldmesser, 2007; Taccone et al., 2015; Kale et al., 2017). In an individual with an impaired immune function, inhaled airborne spores of *A. fumigatus* will not be effectively eliminated and will remain in the airways, causing a range of infections that include allergic bronchopulmonary aspergillosis (ABPA), aspergilloma (chronic aspergillosis), and invasive aspergillosis (IA) (Hohl and Feldmesser, 2007; Van De Veerdonk et al., 2017). Invasive aspergillosis is the most serious infection, with a global prevalence of 250,000 cases per year and mortality rate up to 90–95% (Lin et al., 2001; Maschmeyer et al., 2007).

In addition to the increasing burden of patients with impaired immunity (Hohl and Feldmesser, 2007), another major challenge is the treatment of *Aspergillus* infections due to triazole resistance, the most commonly indicated drugs to treat these infections. Azole resistance occurs due to the presence of the point mutation L98H in the azole target *Cyp51A* and a 34-base pair (bp) tandem repeat (TR34) in its promoter region (Snelders et al., 2009). The most likely accepted cause for the development of azole resistance is the widespread azole-based fungicide use against fungal plant pathogens in agricultural practice (Snelders et al., 2009; Hagiwara et al., 2016; Meis et al., 2016).

Multiple factors drive virulence in *A. fumigatus*, and understanding the mechanisms of host adaptation and evolution of the fungus that promote the establishment of an infection, could help develop novel therapeutic strategies to treat these fungal infections (Askew, 2008). Whole-genome and transcriptome analysis have allowed the discovery and study of new components of *A. fumigatus* biology and pathogenesis. Genomic analyses have identified that *A. fumigatus* contains 8.5% of lineage-specific genes with accessory functions for carbohydrate and amino acid metabolism, transport, detoxification, or secondary metabolite biosynthesis, suggesting that this microorganism has particular genetic determinants that can facilitate an *in vivo* infection (Fedorova et al., 2008).

Virulence of *A. fumigatus* previously assessed in murine infection models using two reference strains Af293 and CEA10, showed a high variability depending on the stimuli used to compromise the immune system (Keller, 2017). However, conclusions of *A. fumigatus* pathogenicity based exclusively on observations from these two reference strains may be biased (Keller, 2017). We categorized *A. fumigatus* isolates into three different groups depending on the source of isolation: (1) environmental, e.g., obtained from decaying vegetation, air samples, or crops; (2) clinical, initially found in patient samples and; (3) experimental, which refers to isolates that were first obtained from a clinical setting, and now used as reference strains (i.e., Af293 or CEA10). Several studies have reported differences in virulence between *A. fumigatus* clinical and environmental isolates, as well as among isogenic isolates, determined by survival tests in animal infection models (Mondon et al., 1996; Alshareef and Robson, 2014; Amarsaikhan et al., 2014; Knox et al., 2016; Ballard et al., 2018). These observations highlight the need to recognize the intraspecies genotypic and phenotypic variation among *A. fumigatus* populations for determining the progression and outcome of the diseases produced by this fungus.

We hypothesized that strains from different sources could possess a different virulence genetic content. To test this hypothesis, we investigated the virulence-related genes (VRGs) of nine *A. fumigatus* isolates, represented by two experimental, five clinical, and two environmental isolates. We screened the genomes of the nine isolates using a database containing 244 *A. fumigatus* VRGs selected from studied literature. As a secondary objective, we analyzed the whole-genome sequences of three clinical isolates, two isolated from the same patient with a fatal IA infection and the other one, a colonizing isolate from another patient, and one experimental strain B5233, generated at the University Medical Center Groningen (UMCG) to identify genomic differences between them. Unlike other studies that define the virulence of *A. fumigatus* using animal infection models, this study uses genomic analysis to assess its virulence potential.

## Materials and Methods

### Background of *A. fumigatus* Isolates

*Aspergillus fumigatus* samples evaluated in this study are summarized in Table 1. Four clinical isolates were included: three isolates (P1MS, P1MR, and P2CS) obtained from the UMCG, Groningen, Netherlands and the strain B5233, kindly provided by the Institute for Disease Control and Prevention of the Academy of Military Medical Sciences, Beijing, China. B5233 is a clinical isolate that demonstrated high virulence in murine infection studies, and it has been used as an experimental strain in *A. fumigatus* pathogenicity studies (Sugui et al., 2007; Jackson et al., 2009). The four isolates were initially identified as *A. fumigatus* by microscopic morphological description and sequencing of the internal transcribed spacer (ITS) region using Sanger sequencing.

**TABLE 1:** Characteristics of *A. fumigatus* isolates investigated in this study.

**Isolate**	**Country**	**Source**	**Resistance**	**Resistance mutation**	**References**
B5233	United States	Clinical/Experimental	Susceptible	^–^	Sugui et al., 2007; Jackson et al., 2009
P1MR	Netherlands	Clinical (UMCG)	Resistant	TR34/L98H	This study
P1MS	Netherlands	Clinical (UMCG)	Susceptible	-	This study
P2CS	Netherlands	Clinical (UMCG)	Susceptible	-	This study
Af293 reference	Unknown	Clinical/Experimental	Susceptible	^–^	Abdolrasouli et al., 2015
12-7505054	United Kingdom	Clinical	Susceptible	^–^	Abdolrasouli et al., 2015
08-12-12-13	Netherlands	Clinical	Resistant	TR34/L98H	Abdolrasouli et al., 2015
08-19-02-30	Netherlands	Environmental	Susceptible	^–^	Abdolrasouli et al., 2015
08-19-02-46	Netherlands	Environmental	Resistant	TR34/L98H	Abdolrasouli et al., 2015

**FIGURE 1:**
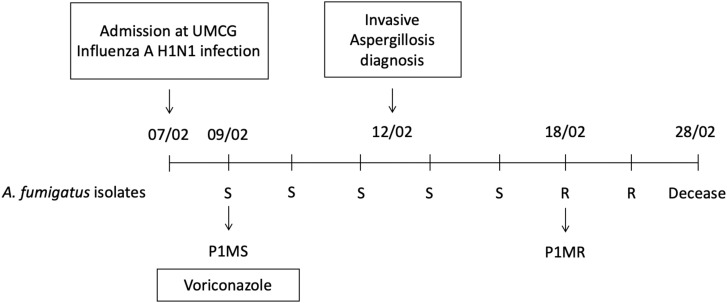
Timeline of the influenza A (H1N1) patient staying in the hospital and the course of infection. A total of seven *Aspergillus fumigatus* isolates were obtained from sputum samples, five susceptible (S) and two resistant (R). The patient remained in the hospital for a period of 21 days until the time of death. Isolates P1MS and P1MR used in this study are indicated in the figure.

P1MS and P1MR, originally isolated from the sputum of the same patient at different time points during a complicated Influenza A (H1N1) virus infection, were considered as mixed-infection isolates, one susceptible and one azole-resistant isolate (Figure 1). This patient was diagnosed with Influenza A (H1N1) virus upon admission and had no other relevant underlying disease. Two days after admission, a positive sputum culture of *A. fumigatus* prompted the initiation of treatment with voriconazole. The patient developed IA at day 5 after admission and passed away 16 days after the diagnosis of the fungal infection. Throughout the course of the IA infection (21 days), a total of seven *A. fumigatus* isolates were recovered, the first five isolates were susceptible to azole treatment and the last two were resistant. We selected the first susceptible and the first resistant isolate to determine their genetic relatedness.

The P2CS isolate was recovered from an individual diagnosed with human immunodeficiency virus (HIV) infection and COPD. The *A. fumigatus* was cultured during a COPD exacerbation event. Doctors discarded chronic pulmonary aspergillosis after a chest imaging study, which did not show the radiological characteristics of pulmonary aspergillosis. Since no indicative symptoms of aspergillosis were identified, the patient was considered as colonized by this strain. The patient was under treatment with antiviral therapy ODEFSEY (emtricitabine/tenofovir alafenamide/rilpitvirine) and treatment for COPD with fluticason, cotrimoxazol, formeterol, and ipratropium.

In addition, the raw sequencing data of five unrelated Dutch and English *A. fumigatus* isolates of environmental and clinical origins (Abdolrasouli et al., 2015), were downloaded from the European Nucleotide Archive (ENA) and included in the study (Table 1).

### Antifungal Susceptibility Testing

The *in vitro* susceptibility of isolates B5233 and P1MS to triazole antifungal drugs was determined using an E-test (AB BIODISK, Solna, Sweden), the agar-based gradient technique for quantitative antifungal susceptibility. The agar-based method VIPcheck^TM^ test (Nijmegen, Netherlands) was used for isolate P2CS, and the susceptibility of P1MR was determined with the *in vitro* broth microdilution reference method from the European Committee on Antimicrobial Susceptibility Testing (EUCAST) (EUCAST, 2019).

### DNA Isolation

Isolates were grown on Potato Dextrose Agar for 7 days at 35°C. The DNA extraction was performed using the DNeasy UltraClean Microbial Kit (Qiagen, Hilden, Germany) according to the manufacturer’s protocol, with some modifications in the initial steps. The initial fungal starting material was obtained using a pre-wetted sterile swab rubbed against the sporulating colony that was dissolved in 700 μl sterile saline solution. The suspension was centrifuged at 10,000 rcf for 4 min. The supernatant was discarded, and the pellet was resuspended in 300 μl of Power Bead solution. This suspension was added to a second pellet of the same sample. The final concentrated suspension was transferred to a Pathogen Lysis Tube L containing beads (Qiagen, Hilden, Germany), 50 μl of solution SL, and 200 μl of sterile saline solution for homogenization. Disruption was carried out in a Tissue Homogenizer Precellys 24 (Bertin, Montigny-le-Bretonneux, France), set to three times at 5,000 rpm for 30 s, and separated by 30 s. The disruption preps were heated to 65°C, as suggested in the Troubleshooting Guide of the protocol, to increase the final DNA yield.

### Library Preparation and Whole-Genome Sequencing

The procedure was performed according to the manufacturer’s protocol (Illumina, San Diego, CA, United States). The fungal genomic DNA (gDNA; 1 ng) of each specimen was used as input DNA for library preparation with NexteraXT DNA Library Prep Kit. Library quality was determined by measuring the fragment size on a 2200 TapeStation System with D5000 Screen tape (Agilent Technologies) and quantified with Qubit 2.0 Fluorometer using Qubit dsDNA HS Assay Kit (Life Technologies, Thermo Fisher Scientific, Waltham, MA, United States). NexteraXT libraries were denatured and diluted to the required molarity for the Illumina sequencing platform and then, two pools were made containing each two libraries. Whole-genome sequencing was performed in two separate runs using the MiSeq Reagent Kit v2 500-cycles Paired-End on a MiSeq Sequencer (Illumina).

### Quality Control and *de novo* Assembly

The raw sequencing reads were quality trimmed using the CLC Genomics Workbench software v10.1.1 with default settings, except for the modification where ‘trim using quality scores was set to 0.01.’ The quality of the nine *A. fumigatus* genome assemblies is shown in Supplementary Table 1. *De novo* assembly produced acceptable results that surpassed >100 coverage with >90% of reads used.

### Identification of Virulence-Related Genes

We considered the review on genes and molecules involved in IA by Abad et al. (2010), the online gene database AspGD^1^, and bioactive secondary metabolite genes encoding Biosynthetic Gene Clusters (BGCs) 3, 5, 6, 14, 15, and 25, as part of the pathogenic arsenal of this fungus (Bignell et al., 2016; Kjaerbølling et al., 2018) to build a comprehensive database. The 244 VRGs included in the database could be categorized into seven groups according to their involvement in processes, such as thermotolerance, resistance to immune responses, cell wall formation, nutrient uptake, signaling and regulation, and production of toxins and secondary metabolites and allergens (Supplementary Table 2). The *de novo* assemblies of the clinical UMCG isolates and strain B5233 were screened with the ABRicate v0.3 software tool^2^ to detect the presence or absence of VRGs included in the database. The thresholds were set to >90% coverage and >90% identity to determine the presence of a VRG.

### TRESP Genotyping

This method is based on hypervariable tandem repeats located within exons of surface protein coding genes (TRESP) related to cell wall or plasma membrane proteins (Garcia-Rubio et al., 2016). The allele sequence repeats of three TRESP targets, an MP-2 antigenic galactomannan protein (MP2), a hypothetical protein with a CFEM (common in several fungal extracellular membrane proteins) domain (CFEM), and a cell surface protein A (CSP) are combined to assign a specific genotype (Garcia-Rubio et al., 2016). The previously described allele repeats of these proteins were used to Create a Task Template by Allele Libraries in SeqSphere + software v5.1.0 (Ridom GmbH, Münster, Germany) with import option: use as reference sequence ‘best matching allele’ that enabled a dynamic reference sequence. The assembled genomes were imported into SeqSphere + and the specific target repetitive sequences of each protein were analyzed for each UMCG isolate using the ‘find in sequence’ tool to identify the specific allele combination.

### Comparative Genomics

Genome assemblies of UMCG isolates and B5233 were aligned using blast + v2.6 (Camacho et al., 2009), and reads were mapped to the eight reference chromosomal genomes of Af293 (Accession No. NC_007194 -NC_007201) using bowtie2 v2.2.5 (Langmead and Salzberg, 2012). For each contig, local alignment coordinates were extended to their whole length by using the highest bitscore with an in-house script. Mean coverage was calculated every 5 kb using bedtools v2.17 (Quinlan and Hall, 2010). The location of VRGs was determined by local alignment, and GC percentage was calculated every 100 bp with a script^3^. Location and frame of coding sequences were extracted from the reference sequence GenBank files. All gathered information was represented in a circular image using circos v0.69-3 (Krzywinski et al., 2009).

### Identification of Genetic Variants

The variant analysis was performed for the three UMCG isolates, B5233 strain, and two Dutch environmental isolates named 08-19-02-30 and 08-19-02-46. Variants were called against the reference genome *A. fumigatus* Af293 (release 37, FungiDB) using the web-based platform EuPathDB Galaxy Site^4^ (Giardine et al., 2005). The quality control of the raw reads was performed with FastQC (version 0.11.3, Babraham Institute) and trimmed with Sickle (Galaxy version 070113). Trimmed-reads were aligned with the reference using Bowtie2 (Align version 2.1.0 64) (Langmead et al., 2009) and the ‘very sensitive’ alignment default setting. The BAM files were sorted with SAMtools, and variant calling was performed with Freebayes (v0.9.21-19-gc003c1e) and SAMtools (Li et al., 2009). The resulting variants were annotated using SnpEff to predict the impact of a variant on the gene function, classifying them into different categories: high, moderate, low, and modifier^5^ (Cingolani et al., 2012). High impact variants are predicted to have a disruptive effect on the protein (e.g., frameshift variants, inversion), moderate impact variants could change protein effectiveness (e.g., missense variant, in-frame deletion), low impact variants are not expected to have a significant impact on protein function (e.g., synonymous variant), and finally, modifier variants are non-coding changes where predictions are difficult, or there is no evidence of impact (e.g., exon variant, downstream gene variant). SnpSift was used to extract the variants with moderate and high impact by filtering the resulting variant call format (VCF) files from SnpEff (Supplementary Material 5–10).

In addition, identification of single nucleotide polymorphisms (SNPs) present in VRGs of UMCG isolates and B5233 strain was performed using CLC Genomics Workbench software v11.0.1. For this purpose, trimmed-reads of each genome were mapped to a concatenated sequence consisting of 244 VRG genes (Supplementary Table 2). The SNPs were called with a minimum read coverage of 10 and with a minimum frequency of 90%. The VRG sequences used to create the concatenated sequence belonged to the reference *A. fumigatus* Af293.

Snippy v. 4.3.5^6^ was used to determine the number of variants between P1MS and P1MR isolates. The trimmed-reads of P1MR were aligned to the assembly of P1MS for variant calling. In this case, the P1MS draft genome assembly, which is used as the reference, is not annotated, and therefore, a functional prediction of the determined variants was not possible. Accordingly, we only presented a quantitative analysis of the latter.

## Results

### Screening of Virulence-Related Genes

We screened the genome sequences of nine *A. fumigatus* isolates (Table 1) for the presence of particular VRGs using our in-house database (Supplementary Table 2). We identified the presence of all 244 VRGs (>90% coverage and >90% identity) in the genome of seven isolates: P1MR, P1MS, P2CS, Af293, 12-7505054, 08-19-02-30, and 08-19-02-46. In addition, 243 genes were present in the genomes of B5233 and 08-12-12-13, and both isolates lacked the *Afu5g12720* gene. This gene codes for a putative ABC transporter and is a member of the BGC17, consisting of 10 genes (Bignell et al., 2016). The product of this BGC is a non-ribosomal peptide synthetase, thought to have a structural function (O’Hanlon et al., 2011). However, no clear link between this ABC transporter and the function of this peptide has been described before. Therefore, it is unknown how its absence could affect the overall function of this cluster and its specific role in mediating virulence.

### TRESP Genotyping

We used the TRESP genotyping method to determine if the *A. fumigatus* isolates were genetically related. This is especially interesting in the two isolates obtained at different time points from the same patient suffering from an influenza A (H1N1) infection with IA (Figure 1). We wondered whether the susceptible P1MS isolate and the resistant P1MR isolate with 9 days of isolation difference were isogenic, and whether the resistant phenotype developed after azole treatment. The UMCG isolates and B5233 strain presented different allelic combinations, and thus, different TRESP genotypes: P1MS and P1MR having t03m1.1c08A and t11m1.1c09 TRESP genotypes, respectively. In this study, CSP alleles best differentiated the isolates (Table 2).

**TABLE 2:** TRESP genotype based on repetitive sequences in the exons of surface proteins CSP, MP2, and CFEM.

**Sample**	**Allele CSP**	**Allele MP2**	**Allele CFEM**	**TRESP genotype**
B5233	t02	M1.2	c09	t02m1.1c09
P1MR	t11	M1.1	c09	t11m1.1c09
P1MS	t03	M1.1	c08A	t03m1.1c08A
P2CS	t02	M1.1	c19	t02m1.1c19

### Comparative Genomics

**FIGURE 2:**
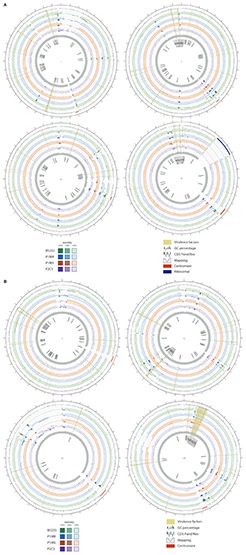
Graphical representation of assemblies and reads of B5233 (green), P1MR (blue), P1MS (orange), and P2CS (purple) isolates aligned to Af293 reference chromosomes: I, II, III, IV **(A)**; V, VI, VII, VIII **(B)**. Outer track indicates all CDS in forward (dark gray) or reverse (light gray) strand. Two different tracks are represented per isolate: one corresponding to the mapping coverage and another one corresponding to contig alignment (minimum ID 85%). The complete contigs are represented with transparency in accordance to the local alignment identity. Genes related to virulence are highlighted in yellow with its names in the innermost track. GC% is represented every 100 bp. Red lines indicate putative centromeres and the dark blue line (chromosome 4) represents the ribosomal DNA.

We compared the genomes of our UMCG isolates and B5233 strain with the *A. fumigatus* Af293 chromosomes. The comparison of the genomic sequences of the eight chromosomes is shown in Figure 2; the researched VRGs locations are highlighted in yellow. We observed small deletions (100 kbp) at the end of chromosomes 5 and 6, and large deletions (>300 kbp) at the beginning of chromosome 1 and at the end of chromosome 7. Multiple small deletions and large-scale deletions in *A. fumigatus* genomes have been reported, and particularly, the large-scale deletions were previously described in chromosome 1 (Abdolrasouli et al., 2015; Garcia-Rubio et al., 2018) and chromosome 7 (Garcia-Rubio et al., 2018). A region with a high dissimilarly, ranging from 1,698 to 2,058 kbp, compared to the reference Af293 is observed in chromosome 7 for all the isolates, except for P2CS that had a certain degree of similarity (Figure 2). Sequence gaps with no assigned CDS represent: (i) putative centromeres in all chromosomes (indicated by a red line in Figure 2), and (ii) a region of ribosomal DNA in the chromosome 4 (indicated by a dark blue line in Figure 2) (Fedorova et al., 2008). We observed repeat-rich sequences in chromosomes 1, 2, 4, 6, and 8, represented by the alignment of many small contigs that coincides with a low GC content (Figure 2). In the case of chromosome 4, a group of VRGs appears to be flanked by these repetitive regions on both sides, whereas some groups are only flanked on one side as depicted in chromosomes 6 and 8 (Figure 2).

### Genomic Variability Among the Fungal Genomes

Variant calling using the *A. fumigatus* Af293 genome as reference identified a total number of 68,352; 48,590; 56,362; and 56,422 variants in the genome of B5233, P1MS, P1MR, and P2CS isolates, respectively (Table 3). High and moderate impact variants were retrieved, and their predicted effect is displayed in Supplementary Table 3. Among the predicted moderate- and high-impact variations, a high number of missense variants ranging from 9,804 to 12,067 were identified that could affect the gene function (Supplementary Table 3). The SNP analysis in VRGs with respect to the reference Af293 strain revealed the presence of SNPs in the range of 1,015–1,122 in all the analyzed isolates (Table 3). Examples of some variants present in the VRGs are listed in Table 4, and a more detailed description is given in Supplementary Table 4. We did not observe any distinct pattern of variant distribution among the VRGs, and thus, we could not assign a particular variant profile based on the origin of the isolate (Table 4). Instead, we observed some cases where all the isolates had the same SNPs in the same gene as demonstrated for *thtA, sidC*, and *msdS* genes. Genes associated with resistance to the immune response, such as *rodB*, *cat1*, and *afpmt2* had only one or no variants, suggesting that they are highly conserved genes. The *gliZ* gene, required for the regulation of gliotoxin and the *gli* cluster, as well as the *sidC* gene, with an essential role in iron acquisition, are examples of genes with different variants in the studied isolates.

**TABLE 3:** Variant analysis of the novel *A. fumigatus* isolates against reference *A. fumigatus* Af293.

	**SnpEff**	**SnpSift filter**		**SNPs present in the virulence genes (CLC Genomics Workbench)**
			
**Isolates**	**Total number of variants**	**Moderate**	**High**
B5233	68,352	12,085	884	1122
P1MS	48,590	10,109	752	1107
P1MR	56,362	11,718	870	1015
P2CS	56,422	12,085	884	1158

**TABLE 4:** Examples of shared and unique moderate and high impact variants in genes associated to thermotolerance, resistance to the immune response, cell wall formation, nutrient uptake, and production of toxins and secondary metabolites and allergens.

		**Per strain**					
**Gene**	**Conserved in all**	**B5233**	**P1MR**	**P1MS**	**P2CS**	**08-19-02-30**	**08-19-02-46**
*thtA*	c.3698T > C c.3458T > C	c.3277C > T	–	c.2828C > T c.2188C > T c.1010G > T	c.3277C > T c.-598G > A	c.272C > A c.2128C > T HIGH stop gained p.Arg710*	–
*pmt1*	–	–	c.442G > A	–	–	–	–
*rodB*	–	–	–	–	–	–	–
*cat1*	–	–	–	–	–	–	–
*catA*	–	–	c.1385G > A	c.1385G > A c.982G > A	–	–	–
*afpmt2*	–	–	–	–	–	–	–
*laeA*	–	–	–	c.189G > A HIGH stop codon gained p.Trp63* c.400C > T	–	–	–
*gliZ*	–	c.1177C > G c.425_427delCAA disruptive_in frame_ deletion p.Thr142del	c.79A > G c.99_101dupTGC c.388A > G c.405_406insACAACAACAACA c.406_409delGCAGinsACAA c.464T > G c.612C > T c.718T > G c.1087T > C	c.388A > G c.464T > G c.1087T > C c.397_409delGCAGCAGCAGCA GinsACAACAACAACAAAAACAA missense_variant&disruptive _inframe_insertion	c.388A > G c.464T > G c.1087T > C c.99_101dupTGC disruptive_ inframe_insertion c.411_412insGCAACAACA c.412A > G	c.388A > G c.464T > G c.397_409delGCAGCAG CAGCAGinsACAACAAC AACAAAAA missense_variant&disruptive_ inframe_insertion c.1338_1340delCTC disruptive_inframe_deletion c.1435A > G	c.1177C > G c.412A > G c.406_411dupGCAGCA c.415A > G c.1404A > C
*msdS*	c.295T > C HIGH stop_lost p.Ter99Glnext*?	c.328C > G c.208T > A		c.328C > G c.208T > A	c.328C > G c.208T > A		c.328C > G c.208T > A c.1043C > T
*sidC*	c.3391A > G c.9598G > A c.9727T > C c.11935T > G c.14222G > A	c.577A > G c.1569C > G c.2311G > A c.3820A > G c.7174A > G c.11326A > G	c.577A > G c.1569C > G c.2311G > A c.3820A > G c.7174A > G c.3401C > T c.13019A > T	c.751C > G c.13019A > T	c.878C > T c.11341C > T c.11935T > G	c.1781T > C c.4771C > A c.5798T > C	c.577A > G c.1569C > G c.2311G > A c.3820A > G c.7174A > G c.9769C > A c.13067A > C

*^∗^ = Stop codon gained.*

Additionally, we performed a comparative analysis between P1MS and P1MR isolates from the same patient and detected 45,335 variants, corresponding to 38,319 SNPs; 868 multiple nucleotide polymorphisms (MNPs); 1,768 insertions; 1,842 deletions; and 2,538 complex mutations (a combination of SNPs and MNPs).

## Discussion

*Aspergillus fumigatus* is a major fungal pathogen capable of causing chronic and deadly invasive infections. Here, we performed a genomic analysis to investigate the virulence potential of this pathogen at the genomic level. We hypothesized that *A. fumigatus* isolates recovered from a patient who died after infection with influenza A (H1N1) and IA, and an isolate from a patient with HIV and COPD with no reported *Aspergillus* infection would reveal a distinct virulence genomic content. In addition to our clinical isolates, we studied the known virulent *A. fumigatus* experimental strain B5233 and the genomes of five unrelated isolates available in a public database, their source of isolation being different (Table 1). Our analysis identified 244 VRGs in all tested *A. fumigatus* isolates, with the exception of *Afu5g12720* gene in B5233 and 08-12-12-13 genomes, indicating that all the studied isolates had the genetic information for virulence. These results suggest that differences in the *A. fumigatus* virulence capacity may not be determined by the presence or absence of virulence factors at the genomic level. This finding is in concordance with the development of an *Aspergillus* infection depending primarily on the alteration of the host immune status. Moreover, the high variability in the studied *A. fumigatus* genomes reflects the enormous capacity of the fungus to adapt to different environments.

Amongst the 244 VRGs included in our in-house database, the *Afu5g12720* gene was the only gene that was undetected in B5233 and 08-12-12-13 isolates. This gene was reported to be absent in 21 out of 66 *A. fumigatus* samples in a population genomics study that investigated the genomic variation of secondary metabolites in this species (Lind et al., 2017). This gene is a member of the BGC17, and its absence could have a functional impact on the synthesis of the final product of this cluster, a non-ribosomal peptide synthetase, which is thought to have a structural function (O’Hanlon et al., 2011). The *Afu5g12720*, coding for an ABC transporter is located in the BGC17 along with other nine genes (Bignell et al., 2016) and was absent in B5233, an experimental strain that has been described as highly virulent. It would be interesting to further study the link between the lack of this gene and a possible increase in virulence, since disruption of another gene member of BGC17, *pes3*, resulted in a hypervirulent strain (O’Hanlon et al., 2011).

The comparative genomic analysis provided additional information about changes in the genome structure of our isolates. We observed an absence of segments at the beginning of chromosome 1 and at the end of chromosomes 5, 6, and 7 in isolates B5233, P1MS, and P1MR when compared with the reference strain Af293. Fedorova et al. (2008) described these segments as subtelomeric regions enriched for the presence of pseudogenes, transposons, and other repetitive elements. Previous reports have suggested that these genes have most likely evolved from big duplication and diversification events and not from horizontal gene transfer (Fedorova et al., 2008). Likely, these segments are insertion-prone regions that contribute to the diversification of the species.

The nucleotide variant analysis of four isolates sequenced at the UMCG identified 48,590 to 68,352 variations compared with the reference strain Af293. This range is similar to the previously reported genetic diversity for *A. fumigatus* determined in 95 sequences, ranging from 36,000 to 72,000 SNPs (Knox et al., 2016). The large number of identified variants and differences in the genome structure displays a broad genetic diversity in the studied isolates. This diversity is hypothesized to directly influence the virulence of the fungus by allowing adaptation to the host environment, the evasion of the host immune system, and the acquisition of antifungal resistance (Rizzetto et al., 2013; Hagiwara et al., 2014; Verweij et al., 2016; Ballard et al., 2018). The presence of SNPs in the VRGs of the clinical isolates, particularly those predicting high impact variations, could be of major influence in the virulence of these isolates. However, we could not link the presence of nucleotide changes in VRGs with a specific origin of isolation. In addition, some repetitive elements were located on the sides of some groups of VRGs, as exemplified by chromosomes 6 and 8. These repetitive sequences could play a role in the expression of these genes since they are recognized to shape fungal genomes (Muszewska et al., 2017). Follow-up studies using RNA sequencing could help elucidate the expression of these virulence genes as well as determine the impact of genomic variations on expression levels. Subsequent infection model studies in animals could be used to correlate these genomic variations and changes with specific pathogenic phenotypes.

The genome sequence of isolates P1MS and P1MR differed by 45,335 variants, and they had different TRESP genotypes, indicating the presence of different *A. fumigatus* isolates with different azole susceptibility profiles in the same patient. It is unlikely that the susceptible isolate would have been able to mutate and acquire azole resistance in a period of 9 days since the median time of development of azole resistance has been reported to be 4 months (Camps et al., 2012). Moreover, the emergence of the resistant phenotype within the host is observed in chronic infections, whereas the acquisition of resistance during IA continues to be unreported (Verweij et al., 2016). However, our current approach cannot determine if the resistant isolate co-existed with the susceptible population since the beginning, or if the resistant isolate was newly acquired during the hospital stay.

In a similar case of post-influenza aspergillosis, four *A. fumigatus* isolates were obtained from a patient that received an allogeneic stem cell transplant and developed IA after the influenza virus infection, which was initially treated with voriconazole (Talento et al., 2018). The first three isolates were susceptible to azole treatment, while the last one exhibited triazole resistance. The resistant isolate differed from the initial isolates as confirmed by STR*Af* microsatellite genotyping (Talento et al., 2018).

The most plausible hypothesis is that the resistant *A. fumigatus* isolate, both in our study and the post-influenza study (Talento et al., 2018), was of environmental origin and that it co-existed with the susceptible isolates in a mixed population that was not detected during the first sampling. Treatment with voriconazole most probably eradicated the initial susceptible strain, and through selective pressure, allowed the resistant *A. fumigatus* strains to persist in the patient’s airways. The possibility of an initial mixed population led to a change in the method of *A. fumigatus* isolation at the diagnostics laboratory at the UMCG; antifungal susceptibility testing is now applied to at least five colonies obtained from a single respiratory sample. Previous studies have reported that influenza infections alter the host immune response, favoring an *Aspergillus* co-infection (Lee et al., 2011; Ghoneim et al., 2013; Crum-Cianflone, 2016). Recently, influenza virus infection has been described as a clear independent risk factor for invasive pulmonary aspergillosis. Therefore, extreme care is advised for patients admitted into the ICU with severe influenza virus infection (Schauwvlieghe et al., 2018).

In this study, TRESP genotyping indicated that the isolates were genetically unrelated. This genotyping method was easy and accessible, and only required the whole-genome sequence of the isolates in contrast to other traditional typing methods, such as MLST, with a lower discriminatory power (Vanhee et al., 2009), the laborious microsatellite determination method (STR*Af*) (Klaassen and Osherov, 2007), or the novel whole-genome SNP-based typing method, which is highly dependent on the variant calling parameters and selection of a genetically close reference strain (Garcia-Rubio et al., 2018).

Our results are in agreement with the hypothesis that the basis of *A. fumigatus* virulence is provided by the evolution of the distinct mechanisms of stress resistance, but lacks dedicated virulence factors, in contrast to bacterial pathogens (Mccormick et al., 2010; Rizzetto et al., 2013). To define the virulence of an *A. fumigatus* isolate, many researchers have characterized different aspects of the fungus, such as the differences in the colonial and spore color phenotype (Rizzetto et al., 2013), the strain-dependent immunomodulatory properties induced in the host (Rizzetto et al., 2013), the clinical or environmental source of the isolate (Mondon et al., 1996; Rizzetto et al., 2013; Kowalski et al., 2016), the ability to adapt and grow in stressful conditions such as low oxygen microenvironments where hypoxia fitness strongly correlated with an increase in virulence (Kowalski et al., 2016), and the ability of the fungus to adjust its gene expression to survive in different immunosuppressive conditions inside the host (Kale et al., 2017). Further research on the virulence of this microorganism should take into consideration all these aspects to determine their infectivity. The results can be used to explore the link between the virulent phenotype and genotype to understand the mechanisms of infection of this pathogen.

This study has some limitations to be considered. First, the number of isolates was small, although three different *A. fumigatus* population sources (clinical, environmental, and experimental) were included. Nevertheless, our findings should be investigated in a larger population to fully corroborate the observation that all members of this species are potentially pathogenic at the genetic level. Second, we included 244 genes in our in-house database based on the current knowledge of *Aspergillus* virulence, but we do not rule out the possibility that other genes may be related to virulence.

## Conclusion

We developed an in-house database with 244 VRGs and detected all of them, except *Afu5g12720*, in the whole-genome sequence of five clinical, two environmental, and two experimental *A. fumigatus* isolates. We did not observe any association between a virulence genetic content and an isolate of specific origin. Moreover, a broad genomic variability and the convenient location of transposable elements that are known to shape the genome reflects the adaptability of *A. fumigatus*, which challenges the development of effective treatments and specific diagnostic tools. Understanding the expression mechanisms of the VRGs may ultimately explain the regulation of the virulence of *Aspergillus* and help improve the handling of *A. fumigatus* infections.

## Data Availability

The sequence raw reads generated in this study have been submitted to the European Nucleotide Archive under project accession number PRJEB28819. Variant analysis results are available as Supplementary Material.

## Ethics Statement

The fungal isolates used for the present analyses were collected in the course of routine diagnostics and infection prevention control. Oral consent for the use of such clinical samples for research purposes is routinely obtained upon patient admission to the UMCG, in accordance with the guidelines of the Medical Ethics Committee of the University Medical Center Groningen. All experiments were performed in accordance with the guidelines of the Declaration of Helsinki and the institutional regulations, and all samples were anonymized.

## Author Contributions

FP-B, SG, MC, and CO designed the experimental set-up. SG, MC, JR, and MS supervised the study. FP-B, ER, and MP performed the experiments. FP-B, SG, MC, and PS-C analyzed the data. SG, JR, MS, LH, and MC provided constructive criticism on the writing. FP-B wrote the manuscript. All authors reviewed the manuscript.

## Conflict of Interest Statement

JR consults for IDbyDNA. The remaining authors declare that the research was conducted in the absence of any commercial or financial relationships that could be construed as a potential conflict of interest.

## Footnotes

1 http://www.aspgd.org/

2 https://github.com/tseemann/abricate

3 https://github.com/DamienFr/GC-content-in-sliding-window-

4 https://eupathdb.globusgenomics.org/

5 http://snpeff.sourceforge.net/SnpEff_manual.html

6 https://github.com/tseemann/snippy
