# Supplementary material for: Revealing the Virulence Potential of Clinical and Environmental Aspergillus fumigatus Isolates Using Whole-Genome Sequencing
Source: Front Microbiol. 2019 Sep 4;10:1970. doi: 10.3389/fmicb.2019.01970 (PMC6737835; doi:10.3389/fmicb.2019.01970)
Supplement: Supplementary file 7 [file Table_1.DOCX]

# Supplementary Data

| **Supplementary Table 1.** Summary of d*e-novo* assemblies using CLC Workbench v10.1.1. | | | | | | | |
| --- | --- | --- | --- | --- | --- | --- | --- |
| **Isolates** | **Read count (millions)** | **Number of contigs** | **N50** | **Max contig length** | **Contig total bp** | **Coverage** | **% reads used** |
| B5233 | 17.2 | 678 | 96,431 | 493,207 | 28,104,988 | 103.6 | 93.8 |
| P1MR | 18.4 | 681 | 87,731 | 357,618 | 28,380,688 | 108.3 | 93.8 |
| P1MS | 20.8 | 575 | 121,650 | 481,048 | 27,949,900 | 153.6 | 97.0 |
| P2CS | 14.9 | 732 | 90,374 | 404,347 | 28,782,304 | 108.5 | 99.9 |
| Af293 reference | 49.8 | 279 | 393,523 | 2,060,164 | 28,607,015 | 166.9 | 98.6 |
| 12-7505054 | 53.4 | 292 | 340,766 | 1,344,010 | 28,085,582 | 181.5 | 98.6 |
| 08-12-12-13 | 36.9 | 295 | 382,782 | 1,005,642 | 28,048,921 | 126.4 | 98.9 |
| 08-19-02-30 | 47.2 | 199 | 525,923 | 1,051,635 | 28,459,975 | 159.2 | 98.9 |
| 08-19-02-46 | 52.1 | 313 | 370,595 | 1,806,848 | 28,341,050 | 175 | 98.6 |

**Supplementary Table 2.** Virulence related genes included in our in-house database for the screening of *A. fumigatus* isolates*.*

| **Function** | **Gene ID** | **Gene Name** | **Product Description** |
| --- | --- | --- | --- |
| **Thermotolerance** | Afu1g03992 | *thtA* | Thermotolerance protein, essential for growth at high temperatures |
|  | Afu3g06450 | *pmt1* | Protein O-mannosyltransferase, required for heat resistance, cell wall integrity, and normal conidiation and conidial germination |
|  | Afu5g04170 | *hsp90* | Heat shock protein |
|  | Afu8g02750 | *cgrA* | Nucleolar rRNA processing protein |
| **Resistance to immune response** | Afu1g03200 | *mfsC* | Putative major facilitator superfamily (MFS) transporter |
|  | Afu1g10380 | *nrps1* | Non-ribosomal peptide synthetase (NRPS) |
|  | Afu1g10390 | *abcB* | Putative ABC multidrug transporter |
|  | Afu1g12690 | *mdr4* | ABC multidrug transporter |
|  | Afu1g13330 | *arp2* | Ortholog(s) have ATP binding, ATPase activity |
|  | Afu1g14330 | *abcC* | Putative ABC transporter |
|  | Afu1g14550 | *sod3* | Putative manganese superoxide dismutase |
|  | Afu1g15490 | *mfsB* | Putative major facilitator superfamily (MFS) transporter |
|  | Afu1g17250 | *rodB* | Conidial cell wall hydrophobin involved in conidial cell wall composition |
|  | Afu1g17440 | *abcA* | ABC drug exporter |
|  | Afu2g17530 | *abr2* | Laccase abr2 |
|  | Afu2g17550 | *ayg1* | Heptaketide hydrolyase ayg1 |
|  | Afu2g17600 | *pksP* | Conidial pigment polyketide synthase alb1 |
|  | Afu3g02270 | *cat1* | Mycelial catalase |
|  | Afu3g03500 | *mdr3* | Putative multidrug resistance protein |
|  | Afu3g09690 | *catA* | Laminin-binding protein with extracellular thaumatin domain |
|  | Afu3g10830 | *gstA* | Putative glutathione transferase |
|  | Afu3g12120 | *ppoC* | Putative fatty acid oxygenase |
|  | Afu4g00180 | *ppoB* | Fatty acid 8,11-diol synthase |
|  | Afu4g10000 | *mdr2* | ABC multidrug transporter biofilm growth regulated |
|  | Afu4g10770 | *ppoA* | Psi-producing oxygenase A |
|  | Afu4g11580 | *sod2* | Putative manganese-superoxide dismutase |
|  | Afu4g13390 | *arpA* | Ortholog(s) have role in conidiophore development, conidium formation, hyphal growth, nuclear migration along microtubule, regulation of growth rate and cytoplasmic dynein complex, hyphal tip localization |
|  | Afu4g14530 | *tpcF* | Putative theta class glutathione s-transferase |
|  | Afu5g06070 | *mdr1* | ABC multidrug transporter |
|  | Afu5g09240 | *sod1* | Cu/Zn superoxide dismutase |
|  | Afu5g09580 | *rodA* | Conidial hydrophobin |
|  | Afu6g03470 | *fmpD* | ABC transporter fmpD |
|  | Afu6g03890 | *catA* | Spore-specific catalase |
|  | Afu6g04360 | *atrF* | Putative ABC transporter |
|  | Afu6g07210 | *sod4* | Putative copper-zinc superoxide dismutase |
|  | Afu6g09930 | *yap1* | bZIP family transcription factor |
|  | Afu6g12522 | *skn7* | Putative transcription factor and response regulator of a two-component signal transduction system |
|  | Afu7g00480 | *abcE* | Putative ABC transporter |
|  | Afu7g05500 | *gstB* | Putative theta class glutathione transferase |
|  | Afu8g01670 | *cat2* | Putative bifunctional catalase-peroxidase |
|  | Afu8g05710 | *mfsA* | Putative major facilitator superfamily (MFS) sugar transporter |
| **Cell wall** | Afu1g01380 | *och4* | Putative alpha-1,6-mannosyltransferase |
|  | Afu1g04260 | *ENGL1* | Beta-1,3-endoglucanase, associated with cell wall |
|  | Afu1g07690 | *afpmt2* | Protein O-mannosyltransferase |
|  | Afu1g12600 | *chsD* | Putative chitin synthase-like gene with a predicted role in chitin biosynthesis |
|  | Afu1g13280 | *pmi1* | Putative phosphomannose isomerase |
|  | Afu1g15440 | *ags3* | Putative alpha(1-3) glucan synthase |
|  | Afu2g01170 | *gel1* | 1,3-beta-glucanosyltransferase with a role in elongation of 1,3-beta-glucan chains |
|  | Afu2g01450 | *mnn9* | Alpha-1,6 mannosyltransferase subunit with a predicted role in N-linked protein glycosylation |
|  | Afu2g01870 | *chsA* | Putative class I chitin synthase |
|  | Afu2g05150 | *mp2* | Putative glycophosphatidylinositol (GPI)-anchored cell wall protein |
|  | Afu2g05340 | *gel4* | Essential 1,3-beta-glucanosyltransferase, GPI-anchored to the plasma membrane |
|  | Afu2g11270 | *ags2* | Putative alpha(1-3) glucan synthase |
|  | Afu2g12850 | *gel3* | Putative GPI anchored beta(1-3)glucanosyltransferase, belongs to the 7-member GEL family |
|  | Afu2g13440 | *chsE* | Putative class V chitin synthase |
|  | Afu2g15910 | *anp1* | Ortholog(s) have alpha-1,6-mannosyltransferase activity, role in protein N-linked glycosylation and alpha-1,6-mannosyltransferase complex, endoplasmic reticulum localization |
|  | Afu2g17560 | *arp2* | 1,3,6,8-tetrahydroxynaphthalene reductase arp2 |
|  | Afu2g17580 | *arp1* | Scytalone dehydratase arp1 |
|  | Afu3g00910 | *ags1* | Putative alpha(1-3) glucan synthase |
|  | Afu3g06690 | *rho3* | Putative Rho-type GTPase |
|  | Afu3g10340 | *rho2* | Rho-type GTPase |
|  | Afu3g12690 | *glfA* | Putative UDP-galactopyranose mutase, enzyme in the first step of galactofuranose biosynthesis |
|  | Afu3g13200 | *gel6* | Putative beta(1-3)glucanosyltransferase, belongs to the 7-member GEL family |
|  | Afu3g14420 | *chsG* | Putative class III chitin synthase |
|  | Afu4g03240 | *mp1* | Putative cell wall galactomannoprotein |
|  | Afu4g04180 | *chsB* | Putative class II chitin synthase |
|  | Afu4g06820 | *ecm33* | Putative glycophosphatidylinositol (GPI)-anchored cell wall protein with similarity to S. cerevisiae Ecm33p |
|  | Afu5g00760 | *chsC* | Putative class III chitin synthase |
|  | Afu5g02740 | *afmnt3* | Putative alpha-1,2-mannosyltransferase with a predicted role in N-linked protein glycosylation |
|  | Afu5g08580 | *och1* | Putative alpha-1,6-mannosyltransferase that initiates the linkage of the N-glycan outer chain |
|  | Afu5g10760 | *mnt1* | Putative alpha-1,2-mannosyltransferase with a predicted role in protein glycosylation |
|  | Afu5g12160 | *afmnt2* | Putative alpha-1,2-mannosyltransferase with a predicted role in N-linked protein glycosylation |
|  | Afu5g14060 | *rho4* | Putative Rho-type GTPase |
|  | Afu6g06900 | *rho1* | Putative Rho-type GTPase |
|  | Afu6g11390 | *gel2* | GPI-anchored 1,3-beta-glucanosyltransferase |
|  | Afu6g12400 | *fks1* | Putative 1,3-beta-glucan synthase catalytic subunit, major subunit of glucan synthase |
|  | Afu6g12410 | *gel7* | GPI-anchored putative beta(1-3)glucanosyltransferase involved in cell wall maintenance |
|  | Afu6g14040 | *och2* | Putative alpha-1,6-mannosyltransferase |
|  | Afu8g02040 | *och3* | Putative alpha-1,6-mannosyltransferase |
|  | Afu8g02130 | *gel5* | Putative beta(1-3)glucanosyltransferase, belongs to the 7-member GEL family |
|  | Afu8g04500 | *pmt4* | Putative protein O-mannosyltransferase |
|  | Afu8g05630 | *chsF* | putative chitin synthase |
| **Toxins and secondary metabolites** | Afu1g14660 | *laeA* | Protein with similarity to protein methyltransferases, involved in regulation of secondary metabolism |
|  | Afu2g17540 | *abr1* | Multicopper oxidase abr1 |
|  | Afu2g17970 | *fgaFS* | Festuclavine dehydrogenase easG |
|  | Afu2g17980 | *easK* | Cytochrome P450 monooxygenase easK |
|  | Afu2g18000 | *fgaDH* | Chanoclavine-I dehydrogenase easD |
|  | Afu2g18010 | *easM* | Cytochrome P450 monooxygenase easM |
|  | Afu2g18020 | *fgaAT* | Fumigaclavine B O-acetyltransferase easN |
|  | Afu2g18030 | *fgaCat* | Catalase easC |
|  | Afu2g18040 | *dmaW* | Tryptophan dimethylallyltransferase |
|  | Afu2g18050 | *fgaOx1* | FAD-linked oxidoreductase easE |
|  | Afu2g18060 | *fgaMT* | 4-dimethylallyltryptophan N-methyltransferase easF |
|  | Afu3g12900 | *hasB* | Putative transporter |
|  | Afu3g12940 | *hasF* | C6 transcription factor hasF |
|  | Afu3g12950 | *hasG* | FAD-binding domain protein |
|  | Afu4g10460 | *hcsA* | Homocitrate synthase, essential enzyme of the alpha-aminoadipate pathway of lysine biosynthesis |
|  | Afu4g14480 | *tpcL* | emodin anthrone oxidase |
|  | Afu4g14490 | *tpcJ* | Putative dihydrogeodin oxidase |
|  | Afu4g14500 | *tpcI* | Questin oxygenase, putative |
|  | Afu4g14520 | *tpcG* | Monoogygenase tpcG |
|  | Afu4g14540 | *tpcE* | Trypacidin cluster transcription factor |
|  | Afu4g14570 | *tpcB* | Trypacidin synthesis protein B |
|  | Afu4g14580 | *tpcA* | O-methyltransferase tpcA |
|  | Afu4g14770 | *osc3* | oxidosqualene:protostadienol cyclase |
|  | Afu4g14780 | *cyp5081A1* | Putative cytochrome P450 monooxygenase |
|  | Afu4g14790 | *cyp5081B1* | Putative cytochrome P450 monooxygenase |
|  | Afu4g14800 | *sdr1* | Putative short chain dehydrogenase |
|  | Afu4g14820 | *null* | Transferase family protein |
|  | Afu5g12710 | *null* | SET domain protein |
|  | Afu5g12720 | *null* | Putative ABC multidrug transporter |
|  | Afu5g12750 | *null* | hypothetical protein |
|  | Afu5g12760 | *null* | CCCH zinc finger DNA binding protein |
|  | Afu5g12770 | *null* | metallo-beta-lactamase superfamily protein |
|  | Afu5g12780 | *null* | hypothetical protein |
|  | Afu5g12790 | *null* | mitochondrial 3-hydroxyisobutyryl-CoA hydrolase, putative |
|  | Afu6g09630 | *gliZ* | C6 finger domain transcription factor gliZ |
|  | Afu6g09640 | *gliI* | Aminotransferase gliI, putative |
|  | Afu6g09660 | *gliP* | Nonribosomal peptide synthetase gliP |
|  | Afu6g09670 | *gliC* | Cytochrome P450 monooxygenase gliC |
|  | Afu6g09690 | *gliG* | Glutathione S-transferase gliG |
|  | Afu6g09710 | *gliA* | MFS gliotoxin efflux transporter gliA |
|  | Afu6g09720 | *gliN* | N-methyltransferase gliN |
|  | Afu6g09730 | *gliF* | Cytochrome P450 monooxygenase gliF |
|  | Afu6g09740 | *gliT* | Thioredoxin reductase gliT |
|  | Afu8g00190 | *ftmC* | Putative cytochrome P450 |
|  | Afu8g00200 | *ftmD* | O-methyltransferase ftmD |
|  | Afu8g00370 | *fma-PKS* | Fumagillin biosynthesis polyketide synthase |
|  | Afu8g00380 | *fmaC* | Fumagillin biosynthesis acyltransferase |
|  | Afu8g00390 | *fmaD* | Fumagillin biosynthesis methyltransferase |
|  | Afu8g00400 | *null* | Fumagillin biosynthesis methyltransferase |
|  | Afu8g00410 | *metAP* | Methionine aminopeptidase type II |
|  | Afu8g00420 | *fumR* | C6 finger transcription factor fumR |
|  | Afu8g00430 | *null* | hypothetical protein |
|  | Afu8g00440 | *psoF* | Dual-functional monooxygenase/methyltransferase psoF |
|  | Afu8g00460 | *fpaI* | Methionine aminopeptidase type I, putative |
|  | Afu8g00470 | *fmaE* | Antibiotic Biosynthesis Monoxygenase superfamily monooxygenase fmaE |
|  | Afu8g00490 | *Fma-KR* | Stereoselective keto-reductase |
|  | Afu8g00500 | *null* | Putative acetate-CoA ligase |
|  | Afu8g00510 | *fmaG* | Fumagillin biosynthesis cluster P450 monooxygenase |
|  | Afu8g00520 | *fmaA* | Fumagillin biosynthesis terpene cyclase |
|  | Afu8g00540 | *nrps14* | PKS-NRPS hybrid synthetase psoA |
|  | Afu8g00550 | *psoC* | Methyltransferase psoC |
|  | Afu8g00570 | *null* | Putative hydrolase |
|  | Afu8g00580 | *psoE* | Glutathione S-transferase psoE |
| **Allergens** | Afu1g05770 | *exg12* | Secreted beta-glucosidase |
|  | Afu1g06830 | *aspf26* | Putative 60s acidic ribosomal protein superfamily member |
|  | Afu1g09470 | *aspfAT* | Putative class V aminotransferase |
|  | Afu1g14560 | *msdS* | Putative 1,2-alpha-mannosidase |
|  | Afu1g16190 | *aspf9* | Cell wall glucanase |
|  | Afu2g00760 | *aspfPL* | Putative secreted pectate lyase |
|  | Afu2g03720 | *aspf11* | Putative cyclophilin |
|  | Afu2g03830 | *aspf4* | Allergen Asp f 4 |
|  | Afu2g10100 | *aspf8* | Allergen Asp f 8 |
|  | Afu2g11260 | *luA* | Putative 3-isopropylmalate dehydratase with a predicted role in nitrogen metabolism |
|  | Afu2g11850 | *aspf23* | Allergenic ribosomal L3 protein |
|  | Afu2g12630 | *aspf13* | Allergen Asp f 13 |
|  | Afu2g15430 | *AspfSXR* | Sorbitol/xylulose reductase |
|  | Afu3g00590 | *aspHS* | Asp-hemolysin |
|  | Afu3g07430 | *aspf27* | Putative peptidyl-prolyl cis-trans isomerase |
|  | Afu3g14680 | *aspfLPL3* | Putative secreted lysophospholipase B |
|  | Afu4g01290 | *csn* | Glycosyl hydrolase family 75 chitosanase |
|  | Afu4g06670 | *aspf7* | Allergen Asp f 7 |
|  | Afu4g09580 | *aspf2* | Allergen Asp f 2 |
|  | Afu5g02330 | *aspf1* | Allergen Asp f 1 |
|  | Afu5g03520 | *aspfPUP* | Immunoreactive secreted protein |
|  | Afu5g11320 | *aspf29* | Allergen Asp f 29 |
|  | Afu6g02280 | *aspf3* | Allergen Asp f 3 |
|  | Afu6g03620 | *mreA* | FAD/FMN-containing isoamyl alcohol oxidase |
|  | Afu6g04920 | *fdh* | Putative NAD-dependent formate dehydrogenase |
|  | Afu6g06770 | *aspf22* | Putative enolase |
|  | Afu6g10300 | *aspf28* | Allergen Asp f 28 |
|  | Afu7g05740 | *null* | Putative NAD-dependent malate dehydrogenase |
| **Nutrient uptake** | Afu1g01550 | *zrfA* | Putative plasma membrane zinc transporter |
|  | Afu1g09280 | *ptcB* | Putative type 2C protein phosphatase (PP2C) involved in dephosphorylation of SakA MAP kinase in response to osmotic stress |
|  | Afu1g10080 | *zafA* | Putative C2H2 zinc-responsive transcriptional activator |
|  | Afu1g16950 | *pig-a* | Protein required for the initiation of involved in glycosylphosphatidylinositol (GPI)-anchor biosynthesis |
|  | Afu1g17200 | *sidC* | Fusarinine C non-ribosomal peptide synthetase (NRPS), putative |
|  | Afu2g03860 | *zrfB* | Low affinity plasma membrane zinc transporter |
|  | Afu2g04010 | *tpsB* | Putative trehalose-6-phosphate synthase |
|  | Afu2g05730 | *mirC* | Putative siderophore transporter |
|  | Afu2g07680 | *sidA* | L-ornithine N5-oxygenase |
|  | Afu2g08360 | *pyrG* | Orotidine 5'-monophosphate decarboxylase |
|  | Afu2g09030 | *dppV* | Secreted dipeptidyl-peptidase |
|  | Afu3g03400 | *sidF* | Siderophore biosynthesis acetylase AceI, putative |
|  | Afu3g03420 | *sidD* | Nonribosomal peptide synthetase 4 |
|  | Afu3g03640 | *mirB* | Putative siderophore iron transporter |
|  | Afu3g03650 | *sidG* | Putative acetyltransferase with a predicted role in iron metabolism |
|  | Afu3g05650 | *orlA* | Trehalose 6-phosphate phosphatase (T6PP) |
|  | Afu3g09820 | *dvrA* | C2H2 zinc finger domain protein |
|  | Afu3g11400 | *pep2* | Aspartic acid endopeptidase |
|  | Afu3g11970 | *pacC* | C2H2 finger domain transcription factor |
|  | Afu4g07040 | *ctsD* | Putative secreted aspartic-type endopeptidase |
|  | Afu4g08720 | *plb1* | Putative secreted phospholipase B |
|  | Afu4g09320 | *dppIV* | Putative extracellular dipeptidyl-peptidase |
|  | Afu4g09560 | *zrfC* | Zinc transporter that functions in neutral or alkaline environments |
|  | Afu4g11800 | *alp1* | Putative secreted alkaline serine protease |
|  | Afu4g12470 | *cpcA* | Transcriptional activator of the cross-pathway control system of amino acid biosynthesis |
|  | Afu4g13750 | *mep20* | Putative penicillolysin/deuterolysin metalloprotease |
|  | Afu5g01340 | *plb2* | Putative phospholipase B |
|  | Afu5g03790 | *fetC* | Putative ferroxidase |
|  | Afu5g03800 | *ftrA* | Putative high-affinity iron permease |
|  | Afu5g05480 | *rhbA* | Ras-related signaling protein |
|  | Afu5g08570 | *pkaC2* | Class II protein kinase A (PKA) |
|  | Afu5g08890 | *lysF* | Putative homoaconitase |
|  | Afu5g09210 | *alp2* | Autophagic (vacuolar) serine protease |
|  | Afu5g11260 | *sreA* | GATA transcription factor that regulates iron uptake |
|  | Afu5g13300 | *pep1* | Putative extracellular aspartic endopeptidase |
|  | Afu6g01970 | *areA* | Putative GATA-like transcription factor |
|  | Afu6g03590 | *mcsA* | Methylcitrate synthase |
|  | Afu6g04820 | *pabA* | Para-aminobenzoic acid synthetase, an enzyme catalyzing a late step in the biosynthesis of folate |
|  | Afu6g12950 | *tpsA* | Trehalose-6-phosphate synthase |
|  | Afu7g04910 | *Null* | Has domain(s) with predicted hydrolase activity, acting on ester bonds activity |
|  | Afu7g04930 | *pr1* | Putative alkaline serine protease |
|  | Afu7g05930 | *mepB* | Putative metallopeptidase with similarity to mammalian thimet oligopeptidases |
|  | Afu8g02760 | *amcA* | Putative mitochondrial ornithine carrier protein |
|  | Afu8g07080 | *mep* | Putative secreted metalloprotease |
| **Signaling and regulation** | Afu1g05800 | *mkk2* | Putative mitogen-activated protein kinase kinase (MAPKK) |
|  | Afu1g06900 | *crzA* | C2H2-type zinc finger transcription factor involved in calcium ion homeostasis |
|  | Afu1g12930 | *gpaB* | G protein alpha subunit |
|  | Afu1g12940 | *sakA* | Putative mitogen-activated protein kinase (MAPK) with predicted roles in the osmotic and oxidative stress responses |
|  | Afu1g13140 | *gpaA* | G protein-coupled receptor alpha subunit |
|  | Afu1g15950 | *pbs2* | Putative mitogen-activated protein kinase kinase (MAPKK) |
|  | Afu2g00660 | *tcsB* | Putative sensor histidine kinase/response regulator with homology to S. cerevisiae Sln1p |
|  | Afu2g01260 | *srbA* | Sterol regulatory element binding protein (SREBP) |
|  | Afu2g07770 | *rasB* | Ras family GTPase protein |
|  | Afu2g12200 | *pkaC* | cAMP-dependent protein kinase catalytic subunit |
|  | Afu2g12640 | *gprD* | Putative G-protein coupled receptor (GPCR)-like protein |
|  | Afu2g13260 | *medA* | Putative regulator of adherence, host cell interactions and virulence |
|  | Afu3g05900 | *ste7* | MAP kinase kinase (MAPKK) |
|  | Afu3g10000 | *pkaR* | cAMP-dependent protein kinase regulatory subunit |
|  | Afu3g11080 | *bck1* | Putative mitogen-activated protein kinase kinase kinase (MAPKKK) |
|  | Afu3g11250 | *ace2* | C2H2 transcription factor with a role in conidiophore development, pigment production, germination and virulence |
|  | Afu4g13720 | *mpkA* | mitogen-activated protein kinase |
|  | Afu5g06420 | *steC/ste11* | Ortholog(s) have MAP kinase kinase kinase activity, MAP kinase kinase kinase kinase activity, SAM domain binding activity |
|  | Afu5g08420 | *sho1* | Putative transmembrane osmosensor with homology to S. cerevisiae Sho1p |
|  | Afu5g09100 | *mpkC* | Putative mitogen activated protein kinase (MAPK) |
|  | Afu5g09360 | *calA* | calcineurin a catalytic subunit |
|  | Afu5g11230 | *rasA* | Ras family GTPase protein |
|  | Afu5g12210 | *sfaD* | G protein-coupled receptor beta subunit |
|  | Afu6g08520 | *acyA* | Adenylate cyclase of the cAMP-dependent signaling pathway, involved in regulation of proliferation and conidiophore development |
|  | Afu6g10240 | *fos-1* | Putative histidine kinase, two-component signal transduction protein |
|  | Afu6g12820 | *mpkB* | Putative mitogen-activated protein kinase (MAPK) |
|  | Afu7g04800 | *gprC* | Rhodopsin-like G-protein coupled receptor |

*null = no gene name assigned.

| **Supplementary Table 3.** Predicted effect of resulting variants after filtering VCF files with SnpSift. | | | | |
| --- | --- | --- | --- | --- |
| **Impact and predicted effect** | **Isolates** | | | |
|  | **B5233** | **P1MS** | **P1MR** | **P2CS** |
| **High** | 883 | 759 | 877 | 892 |
| Frameshift variant | 346 | 317 | 333 | 333 |
| Splice acceptor | 34 | 24 | 37 | 33 |
| Splice donor | 37 | 29 | 34 | 29 |
| Start lost | 16 | 19 | 19 | 23 |
| Stop gained | 270 | 224 | 269 | 282 |
| Stop lost | 180 | 146 | 185 | 192 |
| **Moderate** | 12,417 | 10,117 | 11,730 | 12,100 |
| Inframe deletion | 173 | 149 | 143 | 168 |
| Inframe insertion | 177 | 164 | 181 | 185 |
| Missense variant | 12,067 | 9,804 | 11,406 | 11,747 |
